# Rab12 Promotes Radioresistance of HPV-Positive Cervical Cancer Cells by Increasing G2/M Arrest

**DOI:** 10.3389/fonc.2021.586771

**Published:** 2021-02-25

**Authors:** Yujie Huang, Yonghao Tian, Wenhao Zhang, Ruijuan Liu, Weifang Zhang

**Affiliations:** ^1^ Department of Microbiology, School of Basic Medical Sciences, Cheeloo College of Medicine, Shandong University, Jinan, China; ^2^ Department of Orthopedics, Qilu Hospital of Shandong University, Jinan, China

**Keywords:** HPV, cervical cancer, radiation, Rab12, G2/M arrest, Cdc2

## Abstract

**Background:**

HPV-positive (HPV+) cervical cancer cells are more radioresistant compared with HPV-negative (HPV-) cervical cancer cells, but the underlying mechanism is not fully illuminated. Our previous mass spectrometry data showed that Ras-associated binding protein Rab12 was up-regulated by HPV, and this study is to investigate the role of Rab12 in the radioresistance of HPV-positive cervical cancer cells.

**Methods:**

CCK-8 assay, colony formation assay, flow cytometry, and Western blot were performed to determine cell proliferation, apoptosis, cell cycle distribution, and protein expressions. DNA damage and repair levels were measured by comet assays and detection of γ-H2AX, XRCC4, and pBRCA1 protein expressions.

**Results:**

Rab12 mRNA and protein expressions were up-regulated in cervical cancer tissues and HPV+ cervical cancer cells. Knockdown of Rab12 enhanced radiosensitivity while overexpression of Rab12 promotes radioresistance. Knockdown of Rab12 alleviated G2/M arrest by decreasing p-Cdc2(Tyr15) after radiation, which was a result of the reduction of p-Cdc25C(Ser216). Rab12 knockdown caused more DNA double-strand breaks (DSBs) and inhibited DNA homologous recombination repair (HRR) after radiation. Instead, overexpression of Rab12 enhanced radioresistance by increasing G2/M arrest, which provided more time for DNA HRR.

**Conclusions:**

Rab12 may serve as a potential therapeutic target to improve clinical treatment outcome of cervical cancer.

## Introduction

Cervical cancer is one of the most common malignancies in women worldwide, and the mortality rate is second only to breast cancer among women aged 20 to 39 years (1). Human papillomaviruses (HPVs) are small DNA viruses that are subdivided into high-risk HPVs (HPV16, HPV18, etc.) and low-risk HPVs (HPV6, HPV11, etc.) according to their pathogenicity (2). Infection of high-risk HPVs is an etiologic factor for the development of cervical cancer, while low-risk HPVs generally cause benign epithelial lesions (3). The HPV encoded oncoproteins E6 and E7 are key viral factors involved in initiation and progression of cervical cancer. HPV E6 protein degrades the tumor suppressor protein p53 (4), and E7 binds and degrades the retinoblastoma suppressor protein pRb (5), inducing increased cell proliferation and genomic instability.

Treatment approaches for cervical cancer include surgery, radiotherapy, and chemotherapy (6). Radiation therapy is an important therapeutic strategy for advanced cervical cancer. Radiation causes DNA damage, especially DNA double-strand breaks, which initiate cell cycle arrest and provide time for the repair of DSBs. Activated p53 induces transcription and activation of p21, leading to inhibition of Cdk4 and Cdk6, which induces G1/S arrest. In addition, p21 binds to and inhibits the kinase activity of Cdc2-cyclin B complex, blocking G2 to M phase transition and causing G2/M arrest (7). However, in HPV+ cervical cancers, these pathways are dysregulated as there is no functional p53.

Ras-associated binding (Rab) proteins are key regulators of vesicles transport, endocytosis and efflux as well as intracellular protein transport and localization (8). More than 60 human Rab proteins have been identified and some Rab proteins are abnormally expressed in many human cancers. Typically, increased expression levels of certain Rabs, such as Rab1, Rab3, Rab5, Rab11, Rab23, Rab27, Rab38, and others in colorectal cancer, brain tumor, breast cancer, lung cancer, gastric cancer, bladder cancer were observed (9, 10). These Rabs exert tumor-promoting properties such as invasion and migration. On the contrary, a minor fraction of Rabs serve as tumor suppressor. For example, Rab17 was decreased in hepatocellular carcinoma (HCC) and overexpression of Rab17 inhibited the tumorigenic properties of HCC cells (11). Interestingly, the same Rab protein may have diverse functions in different types of cancers. For example, Rab25 enhanced the aggressiveness in ovarian and breast cancer cells (12). However, Rab25 was decreased in colon cancer and lower Rab25 expression levels correlated with poor patient prognosis (13). Besides, Rab25 functioned as a tumor suppressor with both anti-invasive and-angiogenic abilities in esophageal squamous cell carcinoma (14).

Rab12 is located on the human chromosome at 18p11.22. Olkkonen et al. showed that Rab12 is associated with the Golgi apparatus (15), but others, including Iida et al. proved that Rab12 is associated with small vesicles in the cytoplasm (16). Recent studies have demonstrated that Rab12 enhances degradation of transferrin receptor (TfR) (17, 18) and the amino acid transporter (PAT4) (19) by controlling the transport of circulating endosomes to lysosomal carriers, indicating that Rab12 indirectly stimulates autophagy (20). Rab12 mRNA levels was significantly up-regulated in cisplatin-resistant gastric cancer cells compared with cisplatin-sensitive cells (21). Mosesson et al. showed that the integrin molecule is transported in the direction of cancer invasion by endocytosis of Rab12 (22). The combination of gene expression analysis and CNA analysis showed that Rab12 was significantly highly expressed in colorectal cancer with lymph node metastasis, suggesting that Rab12 could be a potential oncogene involved in colorectal cancer (23). However, there has been no report of a direct relation between Rab12 and cervical cancer. Our previous mass spectrometry data showed that Rab12 was up-regulated by HPV (data not shown), but the role of Rab12 in cervical cancer is still unclear.

In this study, we explored the mechanism by which Rab12 promotes radioresistance of cervical cancer cells. Studying the role of Rab12 in cervical cancer radiotherapy provide new targets for improving the therapeutic effectiveness of HPV-positive cervical cancers.

## Materials and Methods

### Patients and Tissue Samples

We used 63 cervical cancer tissue samples and 46 non-cancer cervix tissues for mRNA extraction and real-time PCR. All tissue samples were obtained from the Qilu Hospital of Shandong University. Non-cancer cervix tissues were obtained from patients with chronic cervicitis or uterine fibroids who had undergone total hysterectomy, and cancer tissues were derived from patients with cervical cancer after surgery. Experiments were undertaken with the agreement of each patient following acquired informed consent and ethical approval from the Institute of Institutional Research Ethics of Shandong University.

### Cell Culture

Cervical cancer cell lines HeLa, SiHa, CaSki, and C33A were cultured in Dulbecco’s modified Eagle medium (DMEM, Gibco BRL, USA). RPE1 cells were maintained in Ham’s F12 medium and DMEM (DMEM/F12, Gibco BRL, USA) (1:1). RPE1-Vector, RPE1-16E6, RPE1-16E7 cells were selected by culturing with 6.0 μg/ml puromycin (Sigma, USA). HaCaT-Vector, HaCaT-16E7 cells were selected by culturing with 1.0 μg/ml puromycin. The human gastric epithelial immortalized GES-1 cells, gastric cancer HGC-27, MKN45, BGC-823, SGC-7901, and MGC-803 cells, human lung cancer A549, breast cancer MDA-MB-231, osteosarcoma MG63 and colon cancer HCT-8 cells were cultured in RPMI 1640. Human osteoblasts hFOB1.19 cells, University of Michigan Squamous Cell Carcinoma cell lines UMSCC-25, UMSCC-10A and human gastric cancer AGS cells were cultured in DMEM/F12 medium. Human lung cancer NCI-H1975 and osteosarcoma HOS cells were cultured in DMEM. Human osteosarcoma Saos-2 and U2OS cells were cultured in McCoy’s 5A medium. All cells were supplemented with 10% fetal bovine serum (FBS, Gibco BRL, USA), penicillin (100 U/ml) and streptomycin (100 μg/ml)and incubated in a humidified atmosphere of 5% CO_2_ at 37°C. For irradiation, cells were treated with a single dose of 6 Gy X-ray irradiation delivered by a Primus linear accelerator (Siemens, Germany) at a dose rate of 400 cGy/min at the Department of Radiation Oncology, Qilu Hospital of Shandong University.

### Small Interfering RNA Transfection

Specific small interfering RNA (siRNA) targeting 16E6, 16E6E7, 18E6, 18E6E7, and Rab12 and control siRNA were purchased from GenePharma (GenePharma, China). Cells were seeded in 6-well plates and cultured in medium for 24 h before transfection. When cells were 40–50% confluent, the cells in each well were transfected with 20 nM siRNAs using Lipofectamine 2000 (Invitrogen, Life Technologies, USA) according to the manufacturer’s instructions. Cells were harvested for protein knockdown analysis by Western blot. The sequences for siRNA are listed below:

si-Rab12, 5′-GCAUUACCUCAGCUUAUUATT-3′;
si-16E6, 5′-UCCAUAUGCUGUAUGUGAU-3′;
si-16E6E7, 5′-GCACACACGUAGACAUUCG-3′;
si-18E6, 5′-GAGGUAUUUGAAUUUGCAU-3′;
si-18E6E7, 5′-CCUGUGUAUAUUGCAAGAC-3′;
si-NC, 5′-GUAUAUAAGCAAGCAUUAC-3′.

### Establishment of Cell Lines With Rab12 Knockdown and Overexpression

Lentivirus shRNAs were purchased from Vigene Biosciences (USA). To knock down Rab12 gene expression, shRab12 and a negative control were transfected into SiHa cells (Multiplicity of infection, MOI=20) using 5 μg/ml polybrene transfection reagent (GenePharma, China). A concentration of 1 μg/ml puromycin was used to select stably transfected SiHa-shRab12 cells and SiHa-shNC cells.

Adenovirus vector overexpressing Rab12 and negative control were constructed by Vigene Biosciences (USA). C33A cells were seeded at a density of 2×10^5^ cells/well for 24 h. Adenovirus were transfected into C33A cells (MOI=10) using 1×10^-2^ mg/ml ADV-HR (GenePharma, China) transfection reagent. Stably transfected C33A cells expressing pENTER-Control or pENTER-Rab12 were constructed and used for further experiments.

### Western Blot Assay

Total cellular proteins were extracted with radio immunoprecipitation assay (RIPA, Beyotime Biotechnology, China) lysis buffer with phenylmethanesulfonyl fluoride (PMSF, Beyotime Biotechnology, China) and protease inhibitor (BestBio, China) at a 100:1:1 ratio. Protein concentrations were measured by the BCA Protein Assay Kit (Beyotime Biotechnology, China) according to the manufacturer’s instructions. A total of 30 μg of protein was loaded into single well for each condition and was separated by polyacrylamide gel electrophoresis (PAGE). Next, proteins were transferred into PVDF membrane (Millipore, USA). After 1 h blocking with 5% skimmed milk, the membrane was incubated in primary antibody at 4°C overnight. Specific primary antibodies included: Rab12 (1:1,000, 18843-1-AP, Proteintech), p53 (1:1,000, 9282s, Cell Signaling), E2F1 (1:1,000, sc-193, Santa Cruz), GAPDH (1:1,000, AB-P-R001, Hangzhou Goodhere Biotechnology), cleaved Caspase3 (1:1,000, 9661, Cell Signaling), PARP (1:1,000, 9532, Cell Signaling), Cdc25C (1:1,000, 4688, Cell Signaling), p-Cdc25C(Ser216) (1:1,000, 9528, Cell Signaling), Cdc2 (1:1,000, 77055, Cell Signaling), p-Cdc2 (Tyr15) (1:1,000, 9111, Cell Signaling), p-Cdc2 (Thr161) (1:1,000, 9114, Cell Signaling), Cdk2 (1:1,000, 2546, Cell Signaling), Cdk6(1:1,000, 13331, Cell Signaling), cyclin A(1:1,000, 4656, Cell Signaling), cyclin B (1:1,000, 55004-1-AP, Proteintech), cyclin D (1:1,000, 2922, Cell Signaling), phosphor-BRCA1(Ser1524) (1:1,000, 9009, Cell Signaling), XRCC4 (1:1,000, CY1405, Abways). The following day, membranes were washed three times by Tris-buffered saline with Tween 20 (TBST), incubated with the relevant secondary antibody (1:3,000) for 1 h. Immobilon Western Chemiluminescent HRP Substrate (Millipore, USA) was used to display the signals.

### Quantitative Reverse-Transcription PCR (RT-qPCR)

RNA was extracted using TRIzol reagent (Invitrogen, USA) and reverse-transcribed to cDNA by the PrimeScript™ RT Reagent Kit with gDNA Eraser (Takara, Japan) according to the manufacturer’s instructions. SYBR^®^ Premix Ex Taq™ (Takara, Japan) was used to amplify the products and monitored on a DNA Engine Peltier thermal cycler equipped with a Chromo4 real-time PCR detection system (Bio-Rad, USA). PCR primer sequences were as follows:

GAPDH forward, 5′-GCACCGTCAAGGCTGAGAAC-3′;
GAPDH reverse, 5′-TGGTGAAGACGCCAGTGGA-3′;
Rab12 forward, 5’-AGGCCGGCCGACTTCAAGCTG-3’;
Rab12 reverse, 5’-TCAACAGCATCGGACGTGTGG-3’.

### Cell Viability and Cell Proliferation Assays

CCK-8 and colony formation assay were used to detect cell proliferation and viability. For CCK-8 assay, 2×10^3^ cells were plated in 96-well plates and CCK-8 reagent (BestBio, China) was added according to the manufacturer’s protocol, a wavelength of 450 nm was used to measure the absorbance. For colony formation assay, cells were seeded in 6-well plates at 500 cells per well, and medium was changed every 2 days for 2 weeks. Cells were fixed with 4% paraformaldehyde and stained with crystal violet (Beyotime Biotechnology, China) for 20 min.

### Flow Cytometry

The cell cycle was analyzed by flow cytometry. Cells were seeded in 6-well plates and cultured in medium. When cells were 70% confluent, they were trypsinized, washed with PBS, fixed with 70% ethanol, and stored at 4°C overnight. The following day, cells were stained with propidium iodide (PI) in the dark for 30 min at 4°C and analyzed using the CytoFLEX flow cytometer (Beckman, USA). Cell cycle analysis was performed using FlowJo V10 software.

### Apoptosis Assays

Cells in 6-well plates were detached with trypsin and cells were washed with cold PBS. Next, cells were stained with PE Annexin V Apoptosis Detection Kit I (559763, BD PharmingenTM, USA) according to the manufacturer’s protocol. Cell counts were analyzed by CytoFLEX flow cytometry. Apoptosis analysis was performed using FlowJo V10 software.

### Immunofluorescence Staining

Cells were seeded in 24-well plates containing slides. When cells were 60% confluent, the cell slides were removed, fixed in 4% formaldehyde for 15 min, and washed three times. Next, slides were permeabilized with 0.2% Triton X-100 (Sigma, USA) for 30 min and washed with PBS. Mouse anti-γ-H2AX (05-636-AF555, Sigma, USA) was diluted with 2% BSA (1:100), and cell slides were incubated with primary antibody at 4°C overnight. After 24 h, cells were incubated with Alexa Fluor secondary antibody (1:50) in the dark for 2 h. Next, DNA was stained with DAPI (4′,6-diamidino-2-phenylindole, Beyotime Biotechnology, China) for 5 min and then washed with PBST three times. Finally, the cell slides were sealed with glycerin and stored in the dark at 4°C. Slides were observed under a fluorescence microscope.

### Single Cell Gel Electrophoresis (Comet Assay)

DNA damage was assessed by the comet assay. A total of 2×10^5^ cells were suspended in 100 μL 0.5% low melting-temperature agarose and loaded on slides with 0.5% normal melting temperature agarose. Then, slides were incubated in the neutral cell lysate for 2 h at 4°C. The slides were subjected to electrophoresis under neutral conditions at 25V for 25 min, and they were stained using DAPI for 5 min. DNA damage was observed under a fluorescence microscope. Analysis was performed using CASP software.

### Statistical Analysis

Data are expressed as means and standard deviations (SD). All statistical analyses were performed using SPSS version 20.0 (IBM, Chicago, IL, USA). Significant differences between the two groups were assessed by two-tailed Student’s *t*-test. Associations between Rab12 expression and clinicopathological parameters were analyzed using chi-square test. OS were analyzed by the Kaplan-Meier method and compared by log-rank test. A p-values <0.05 was considered statistically significant.

## Results

### Rab12 Was Highly Expressed in Cervical Cancer Tissues and Was Up-Regulated by HPV E6, E7

**Figure 1:**
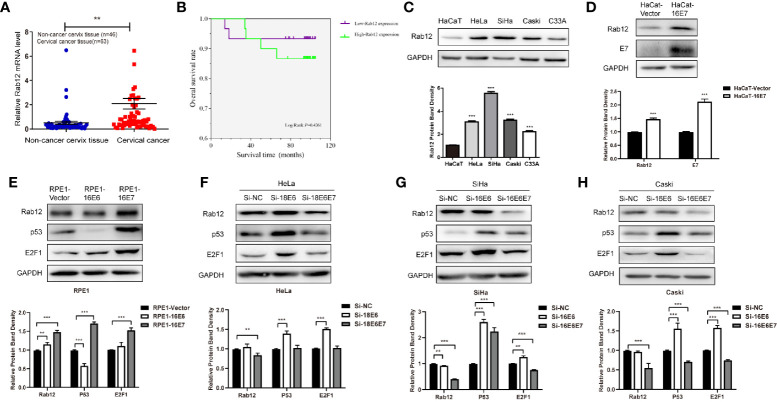
Rab12 was highly expressed in cervical cancer and oncoproteins E6 and E7 promoted the expression of Rab12. **(A)** Real-time PCR showed that Rab12 mRNA level was higher in clinical cervical cancer tissues (n=63) compared with non-cancer cervix tissues (n=46). **(B)** Kaplan-Meier curves for overall survival of 60 patients with cervical cancer according to Rab12 expression (Log-Rank test, P > 0.05). **(C)** Rab12 protein expression was analyzed by Western blot in HaCaT and cervical cancer cell lines HeLa, SiHa, Caski, C33A. **(D, E)** Western blot analysis of Rab12 proteins in HaCaT cells overexpressing 16E7 and Rab12, p53, E2F1 proteins in RPE1 cells overexpressing 16E6, 16E7; GAPDH was used as the loading control. **(F–H)** Protein levels of Rab12, p53, E2F1 in HeLa, SiHa, Caski cells after transfection with siRNA targeting 18E6, 18E6E7, or 16E6, 16E6E7 for 48 h; Comparable Western blots were observed in three independent experiments. Data are presented as mean ± SD from three independent experiments, **p < 0.01, ***p < 0.001.

We first detected Rab12 mRNA expression in cervical cancer tissues. As shown in **Figure 1A**, Rab12 mRNA levels were significantly elevated in cancer tissues (*p<*0.01) compared to non-cancer cervix tissues. The detailed information for patients and disease features were in **Table 1**. However, Rab12 expression was not correlated with age, differentiation status, tumor size, lymph node metastasis, direct metastasis, vascular invasion, histological grade, or clinical stage. To investigate whether Rab12 could be an independent prognostic factor with cervical cancer, we performed survival analysis with overall survival (OS). Overall survival was defined as the time interval between the date of surgery and death from any cause. Follow-up of 60 patients was done, and 54 of them were alive at the end of the follow-up (October 2020). There was no significant difference between the OS rate of patients with high-Rab12 expression and patients with low-Rab12 expression (**Figure 1B**). In addition, we measured Rab12 protein levels in several cervical cancer cell lines using human immortal keratinocytes HaCat cells as a negative control. Rab12 protein was highly expressed in cervical cancer cells. Furthermore, Rab12 levels were higher in HPV+ HeLa, SiHa, and Caski cells than HPV- C33A cells (**Figure 1C**). We also detected the expressions of Rab12 in many other cancer cells and found that Rab12 was abnormally expressed in human osteosarcoma, breast cancer, colon cancer, lung cancer, gastric cancer, and head and neck squamous cell carcinoma cells, which suggested that Rab12 may play different roles in specific cancers (**Supplementary Figure 1**).

**Table 1:** Association of clinicopathological characteristics of patients with cervical cancer and Rab12 expression.

	No. of patients	The expression of Rab12		*P* value
		Low	High	
Age(years)				
≤45	28	17	11	0.941
>45	35	14	21	
Differentiation grade				
Low, moderate	31	29	2	0.668
High	32	29	3	
Tumor size, cm				
≤ 4.0	53	27	26	0.525
>4.0	10	4	6	
LN metastasis				
Yes	19	9	10	0.848
No	44	22	22	
Vascular invasion				
Yes	8	4	4	0.962
No	55	27	28	
Vaginal invasion				
Yes	2	1	1	0.982
No	61	30	31	
Surrounding tissue invasion				
Yes	3	2	1	0.535
No	60	29	31	
Histotype				
AC	8	4	4	0.962
SCC	55	27	28	
FIGO staging				
I	52	23	29	0.086
II	11	8	3	

LN, lymph node; SCC, squamous cell carcinoma; AC, adenocarcinoma; FIGO staging, the International Federation of Gynecology and Obstetrics Staging.

To explore the relationship between HPV and Rab12, HaCaT and RPE1 (human retinal pigment epithelial) cells expressing HPV 16E6 or 16E7 were established using a pBabe retroviral system, as described previously (24). Rab12 was highly expressed in HaCaT-HPV 16E7 (**Figure 1D**), RPE1-HPV 16E6, and RPE1-HPV 16E7 cells (**Figure 1E**) compared with control cells. Additionally, siRNA was used to interfere with HPV 18E6/E7 expression in HPV18+ HeLa cells, and interfere with HPV 16E6/E7 in HPV16+ SiHa and Caski cells. The expression of p53 and E2F1 indicated that E6 or E6E7 expression was knocked down efficiently. The expression of Rab12 was decreased after interfering with 18E6E7 in HeLa cells, but there was no robust change in Rab12 expression after interfering with 18E6 expression (**Figure 1F**). In SiHa and Caski cells, the expression level of Rab12 was markedly decreased after interfering with 16E6E7 expression (**Figures 1G, H**). These data indicated that Rab12 was up-regulated mainly by high-risk HPV E7 protein rather than by E6 protein.

### Radiation Promoted the Expression of Rab12 in HPV+ Cervical Cancer Cells

**Figure 2:**
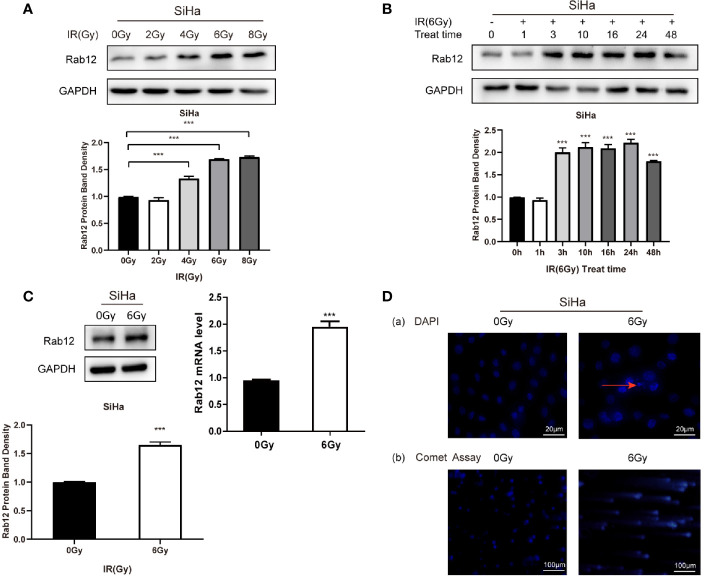
Radiation promoted the expression of Rab12 at both mRNA and protein levels. **(A)** Western blot analysis of Rab12 expression in SiHa cells at different radiation doses (24 h). **(B)** Rab12 expression in SiHa cells at different times after radiation (6Gy). **(C)** Effects of radiation (6Gy, 24 h) on Rab12 expression in SiHa cells were determined by real-time PCR and Western blot analysis. **(D)** Immunofluorescent staining and comet assay showed the presence of nuclear debris and DNA breaks following exposure to 6Gy radiation. Comparable Western blots were observed in three independent experiments. Data are presented as mean ± SD from three independent experiments, ***p < 0.001.

Since the expression of Rab12 was the highest in HPV+ SiHa cells and squamous carcinoma accounts for 80% of cervical cancers, we focused on SiHa cells for further study. We tested the expression of Rab12 at different doses after radiation. As shown in **Figure 2A**, the expression of Rab12 was the highest at 6 Gy, thus, we chose 6 Gy as the experimental radiation dose. We further detected Rab12 protein expression level at different times after 6 Gy radiation, and the results showed that the expression of Rab12 was up-regulated as early as 3 h after radiation and was kept at a high level for up to 24 h (**Figure 2B**). We chose to culture cells for 24 h after radiation and collect the cells for further experiments. We showed that radiation (24 h after 6 Gy) increased the expression of Rab12 at both mRNA and protein levels (**Figure 2C**). In addition, after 6 Gy dose of radiation, DAPI staining showed evidence of nuclear debris and chromatin condensation (**Figure 2D** (a)). Meanwhile, the comet assay showed that the nuclear of the radiological group had a tail, indicating fragmented DNA (**Figure 2D** (b)), which proved that the ray of 6 Gy was effective in inducing DNA damage. In short, radiation promoted the expression of Rab12 in HPV+ cervical cancer cells.

### Knockdown of Rab12 Increased Radiosensitivity while Overexpression of Rab12 Enhanced Radioresistance of Cervical Cancer cells

**Figure 3:**
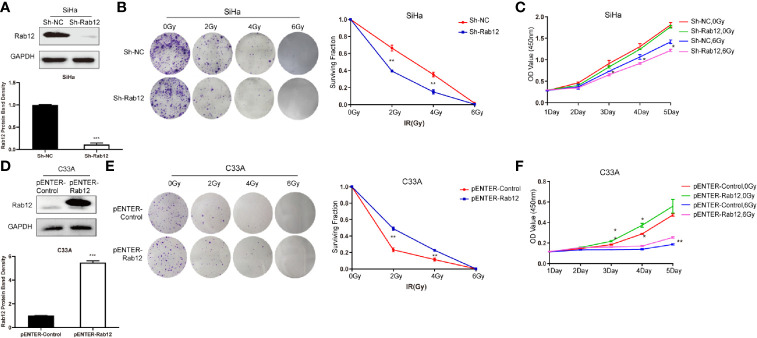
Rab12 affected radiosensitivity of cervical cancer cells. **(A)** Western blot analysis of Rab12 expression after transfection of shRab12 lentivirus. **(B, C)** The effects of radiation on cell proliferation and viability of SiHa-ShRab12 and control cells were detected by the colony formation and CCK-8 assays. **(D)** Western blot analysis of Rab12 expression after transfection of pENTER-Rab12, adenovirus vector overexpressing Rab12. **(E, F)** The effects of radiation on cell proliferation and viability of C33A cells overexpression Rab12 were detected by the colony formation and CCK-8 assays. Data presented as mean ± SD from three independent experiments, *p < 0.05, **p < 0.01, ***p < 0.001.

To test whether Rab12 affected the radiosensitivity of cervical cancer cells, we used lentivirus shRab12 to infect SiHa cells to knock down Rab12 expression (**Figure 3A**). Colony formation and CCK-8 assays were used to detect the effects of radiation on cell proliferation and cell viability (**Figures 3B, C**). The results showed that cells with Rab12 knockdown formed less cell colonies, and the survival fraction and the cell viability were lower compared with control cells after radiation, indicating that knockdown of Rab12 increased the radiosensitivity of HPV+ cervical cancer cells. Additionally, we investigated the effects of Rab12 overexpression (**Figure 3D**) on cell proliferation and viability of C33A cells after radiation. We showed that cells with Rab12 overexpression formed more cell colonies, and the survival fraction and the cell viability were higher than the control cells after radiation (**Figures 3E, F**). Thus, Rab12 enhanced the radioresistance of cervical cancer cells.

### Rab12-Mediated Radioresistance of Cervical Cancer Cells Is Not Due to Inhibition of Apoptosis

**Figure 4:**
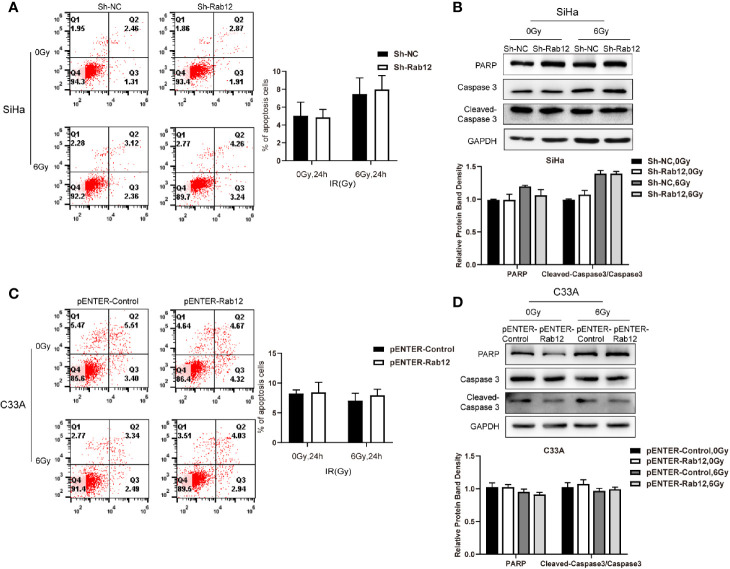
Rab12 exerted no effects on apoptosis of cervical cancer cells after radiation. **(A, C)** Flow cytometry was used to detect the effect of knockdown or overexpression Rab12 on apoptosis 24 h after radiation at 6 Gy. **(B, D)** The expression of apoptosis related proteins PARP, Caspase3, and Cleaved-Caspase3 were detected by Western blot analysis 24 h after radiation at 6 Gy. Data are presented as mean ± SD from three independent experiments.

Next, we investigated whether Rab12 regulated apoptosis of cervical cancer cells after radiation. Interestingly, there were no obvious differences in the percentage of apoptotic cells between Rab12 knockdown SiHa cells and control cells after exposure to radiation (**Figure 4A**). Furthermore, Rab12 knockdown had no effect on the expression of apoptosis-related proteins including PARP and cleaved Caspase-3 (**Figure 4B**). In addition, overexpression of Rab12 in C33A cells had no effect on apoptosis (**Figures 4C, D**). This indicated that Rab12-induced radioresistance was independent of apoptotic activity.

### Rab12 Promoted G2/M Arrest *Via* Up-Regulation p-Cdc2(Tyr15) After Radiation

**Figure 5:**
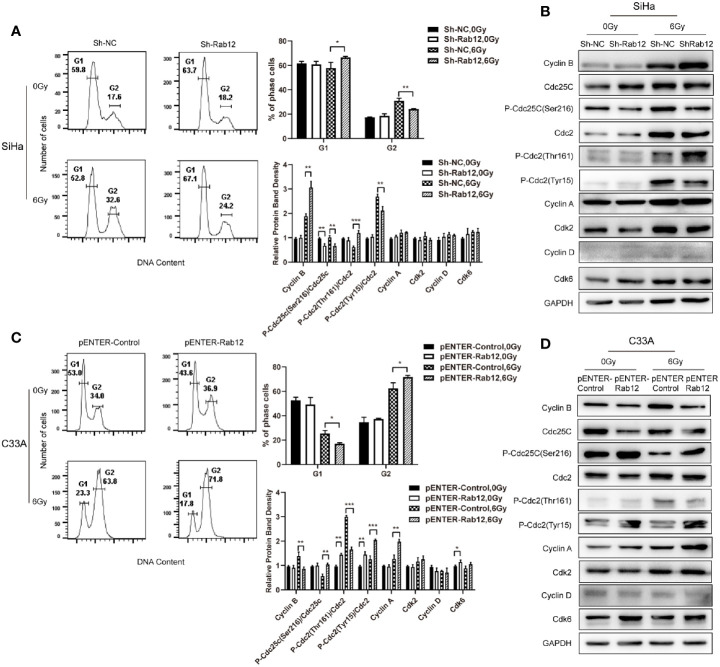
Rab12 induced more G2/M arrest by regulating cell cycle-related proteins after radiation. **(A, C)** Flow cytometry analysis of the effect of Rab12 knockdown and overexpression on cell cycle distribution 24 h after radiation at 6 Gy (stained with PI), G1, G2 phases are indicated; **(B, D)** Cell cycle related proteins before and after radiation in Rab12 knockdown and overexpression cells were detected by Western blot. Comparable Western blots were observed in three independent experiments. Data are presented as mean ± SD from three independent experiments, *p < 0.05, **p < 0.01, ***p < 0.001.

Cell cycle progression determines the rate of cell proliferation, so we explored whether Rab12 influenced the distribution of the cell cycle. Flow cytometry results showed that knockdown of Rab12 did not alter the cell cycle under normal conditions, but the distribution of the cell cycle changed after radiation treatment. Exposure to radiation resulted in a significant increase of cells in the G2/M phase, accompanied with a decrease in the number of cells in the G1 phase. Rab12 knockdown alleviated G2/M arrest caused by radiation (32.6% vs 24.2%, *p* < 0.01) and increased the percentage of cells in the G1 phase compared with control cells (52.8% vs 67.1%, *p* < 0.05) (**Figure 5A**). To understand the underlying mechanism by which Rab12 affected G2/M arrest, the expression of all cell cycle-related proteins were examined. As shown in **Figure 5B**, the steady-state levels of early G1 Cdk-Cdk6 and late G1 Cdk-Cdk2, as well as their partners cyclin D and cyclin A remained fundamentally unchanged in Rab12 knockdown cells and control cells after radiation. However, the expression of cyclin B was increased significantly in Rab12 knockdown cells after radiation. Although the steady-state level of Cdc2 remained unchanged in Rab12 knockdown cells, the inhibitory phosphorylation on Tyr15 residue of Cdc2 was decreased and active phosphorylation on Thr161 was increased after radiation. Since active Cdc25C dephosphorylates Thr14/Tyr15 of Cdc2 while phosphorylation of Cdc25C on Ser216 inactivates Cdc25C, we also detected the expression of Cdc25C and p-Cdc25C(Ser216). We showed that the expression of Cdc25C was unchanged, but the expression of p-Cdc25C(Ser216) was decreased in Rab12 knockdown cells compared with control cells after radiation. These data indicated that knockdown of Rab12 alleviated G2/M arrest by up-regulating cyclin B and p-Cdc2(Thr161) while down-regulating p-Cdc2(Tyr15). Further, the reduction of p-Cdc2(Tyr15) may be due to the decrease of p-Cdc25C(Ser216).

Conversely, overexpression of Rab12 increased G2/M arrest (63.8% vs 71.8%, *p* < 0.05) and decreased the percentage of cells in the G1 phase (23.3% vs 17.8%, *p* < 0.05) after radiation (**Figure 5C**). In addition, the expression of cyclin B was decreased in Rab12-overexpressed cells (**Figure 5D**). The level of Cdc25C and Cdc2 remained unchanged, instead, Rab12 up-regulated p-Cdc25C(Ser216) and p-Cdc2(Tyr15), and down-regulated p-Cdc2(Thr161) protein compared with control cells after radiation, which further enhanced radiation-induced G2/M arrest.

### Rab12 Alleviated DNA Double-Strand Breaks and Promoted DNA Homologous Recombination Repair After Radiation

**Figure 6:**
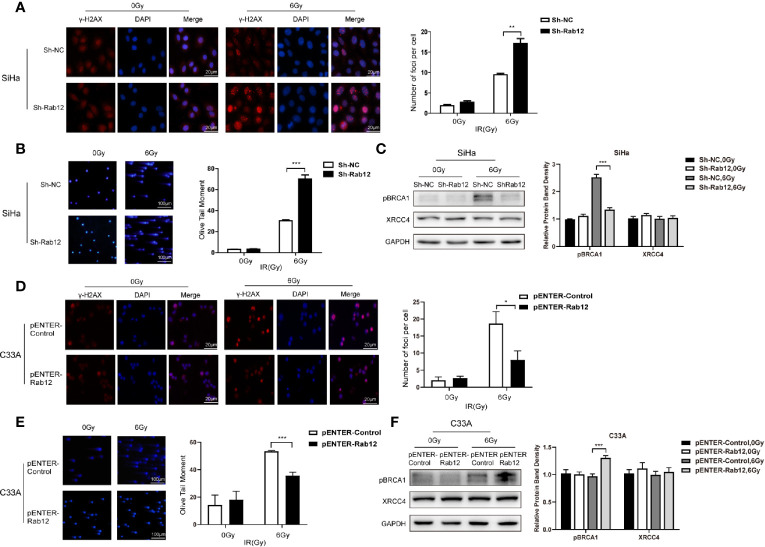
Rab12 alleviated DNA damage after radiation. **(A, D)** Double-strand breaks (DSBs) were analyzed using the neutral comet assay and immunofluorescent staining of γ-H2AX. Immunofluorescence showed that γ-H2AX was located in the nuclear of SiHa and C33A cells (red). There were more γ-H2AX foci in SiHa-ShRab12 cells than control cells, and γ-H2AX foci in C33A-pENTER-Rab12 cells decreased after exposure to radiation than control cells (6Gy, 24 h). DAPI (blue): nuclear counterstain. **(B, E)** The comet assay showed changes in tails in SiHa and C33A cells with Rab12 knockdown or overexpression after radiation. DNA was stained with DAPI (blue). The cells were visualized by fluorescence microcopy. **(C, F)** DSB repair proteins pBRCA1 and XRCC4 in Rab12 knockdown and overexpression cells after irradiation were detected by Western blot. Data are presented as mean ± SD from three independent experiments, *p < 0.5, **p < 0.01, ***p < 0.001.

To further explore the effects of Rab12 on DNA damage and repair, we applied the neutral comet assay and detected immunofluorescence of phosphorylated histone H2AX (γ-H2AX), a marker of DNA DSBs damage. We observed that there were more γ-H2AX foci in the nuclear of Rab12 knockdown cells than control cells after radiation (**Figure 6A**). There was nearly no DNA damage before radiation, but exposure to 6 Gy irradiation exhibited the typical ‘head and tail’ pattern and the average olive tail moment in Rab12 knockdown cells was significantly longer than that in control cells (**Figure 6B**). Conversely, the γ-H2AX foci in the nuclear of Rab12 overexpressed cells were fewer, and the average tail length was shorter than control cells after irradiation (**Figures 6D, E**). In addition, detection of DNA repair-related proteins showed that Rab12 knockdown had no effect on the expression of XRCC4, a non-homologous end joining (NHEJ)-related protein, but inhibited the expression of pBRCA1, a homologous recombination-related protein after radiation (**Figure 6C**). In contrast, overexpression of Rab12 up-regulated the expression of pBRCA1 after radiation (**Figure 6F**). Overall, these data demonstrated that Rab12 alleviated the DNA DSBs and promoted HRR after radiation.

## Discussion

Radiation therapy is an important treatment for advanced cervical cancer. Recurrence and metastasis after radiotherapy are the main causes of treatment failure. In this study, we explored the mechanism of Rab12 in radioresistance of cervical cancer cells. We first verified the effect of HPV oncoproteins E6 and E7 on Rab12 expression by constructing cell lines that overexpress E6/E7 or interfere with E6/E7 expression. Because E6 and E7 proteins are unstable and are easily degraded, we detected the expression of downstream proteins p53 and E2F1 to confirm the expressions of E6 and E7. In RPE1 cells that stably expressed E7, the expression of E2F1 and p53 proteins were increased since E7 degrades pRb protein and releases the transcription factor E2F1. The increase in p53 expression is attributed to the increased expression of E7, although the up-regulated p53 does not play an active biological role (25). The oncoproteins E6 and E7 share the same promoter p97, which is transcribed from a bicistronic sequence, that interferes with E7 expression also inhibits the expression of E6 (26). Therefore, we used interference of E6 as a control to interfere with both E6/E7 expressions. After interference with E6, cells lost the ability to bind and degrade p53, thus p53 expression was increased compared with control cells. After interference with E6/E7, E7 lost the ability to degrade pRb protein and release the transcription factor E2F1, thus E2F1 activity was reduced.

It has been reported that HPV-positive cervical cancer cells SiHa and HeLa are more resistant to radiation than the HPV-negative cervical cancer cell line C33A (27, 28), but the underlying mechanisms are not clear. Our results demonstrated that HPV oncoproteins E6 and E7 up-regulated Rab12 expression, and the expression of Rab12 in HPV-positive cervical cancer cells was higher than in HPV-negative C33A cells. Additionally, we showed that radiation promoted the expression of Rab12, thus we further explored the role of Rab12 in radioresistance of HPV-positive cervical cancer cells.

Compared with other cervical cancer cell lines, the expression level of Rab12 was the highest in SiHa cells; thus, we used a lentivirus to knock down Rab12 expression in SiHa cells to study its effect on the radiosensitivity of cervical cancer cells. Our results showed that cells with Rab12 knockdown formed fewer cell colonies, and both the survival fraction and the cell viability were lower compared with the control cells after radiation, indicating that Rab12 knockdown increased the radiosensitivity of cervical cancer cells. Overexpression of Rab12 in C33A cells further confirmed that Rab12 promoted radioresistance.

It has been reported that ionizing radiation (IR), such as X-rays and gamma (γ)-rays, mediates various forms of cancer cell death such as apoptosis, necrosis, mitotic catastrophe, and senescence. Among these, apoptosis is one of the main mechanisms of IR activity. However, in this study, we showed that the Rab12-induced radioresistance of cancer cells was not related to apoptosis.

Cell cycle arrest promotes DNA repair and maintains genomic stability (29). Cell cycle checkpoints regulate the progression or arrest of the cell cycle in response to DNA damage and allow time for DNA repair. Checkpoints occur either in the late G1 phase, which prevents entry to the S phase, or in the late G2 phase, which prevents entry to mitosis (30). Radiation-induced G2/M arrest is due to activation of the protein kinase, Chk1, which phosphorylates protein phosphatase Cdc25C on Ser216 and inhibits the activity of Cdc25C. Cdc25C dephosphorylates Cdc2 on Thr14/Tyr15 while phosphorylation of Cdc25C prevents the removal of inhibitory phosphates from Cdc2, and the inability to activate Cdc2 results in G2/M arrest (31–33). Our results showed a significant arrest of cells in G2/M phase after radiation. However, knockdown of Rab12 decreased while overexpression of Rab12 increased G2/M arrest compared with control cells after radiation. Active Cdc2-cyclin B complex requires both the dephosphorylation on Thr14/Tyr15, phosphorylation on Thr161 of Cdc2 and increased cyclin B. Our results showed that the knockdown of Rab12 increased the levels of cyclin B. The level of p-Cdc25C(Ser216) was increased after radiation and knockdown of Rab12 down-regulated the expression of p-Cdc25C(Ser216). The decreased p-Cdc25C(Ser216) in cells with Rab12 knockdown indicated more active Cdc25C which dephosphorylated Tyr15 and activated Cdc2. However, how Rab12 regulated the phosphorylation of Cdc25C on Ser216 need to be further explored. We also showed that the phosphorylation of Cdc2 on the Thr161 residue was increased with Rab12 knockdown, which further activated Cdc2. Therefore, elevated cyclin B expression and active Cdc2 promoted the cell transition from the G2 to M phase, which alleviated the G2/M arrest. Overexpression of Rab12 in C33A cells induced further G2/M arrest after radiation by down-regulating cyclin B expression and inhibiting the activity of Cdc2. We hypothesized that the increase in G2/M arrest regulated by Rab12 provided more time for DNA repair.

Following exposure to ionizing radiation, cells undergo cell cycle arrest to repair DNA damage. The repair of double-strand breaks in eukaryotic cells is resolved by two distinct mechanisms: DNA homologous recombination repair during the S and G2 phases and non-homologous end joining throughout the cell cycle (34). Previous reports have shown that γ-H2AX is a novel biomarker for DNA DSBs, which is a potential molecular target to enhance the effects of radiotherapy (35, 36). In our study, overexpression of Rab12 alleviated the DNA DSBs and up-regulated HRR-related protein pBRCA1 after radiation, which may promote the HRR pathway. Although overexpression or knockdown of Rab12 did not change the expression of XRCC4, this does not necessarily prove that cells are not utilizing NHEJ. Examination the subcellular localization of XRCC4 to test whether XRCC4 is recruited to DSBs and a NHEJ reporter assay to test DNA repair activity may be needed. In addition, other error-prone DSB repair pathway, namely alternative end joining (alt-EJ) has been recently shown to repair DSBs in many different conditions. Studies in mammalian cells showed that, unlike NHEJ which requires XRCC4/DNA ligase IV (LIG4) complex activity, XRCC1/DNA ligase III (LIG3) complex were usually involved in alt-EJ pathway (37). Besides, we showed that overexpression of Rab12 decreased γ-H2AX foci and olive tail moment after radiation. Therefore, Rab12 induced radioresistance partly by promoting the HRR pathway.

In conclusion, our results demonstrate that Rab12 is highly expressed in cervical cancer tissues and HPV+ cervical cancer cell lines. Further, the HPV oncoproteins E6 and E7 up-regulate the expression of Rab12, which induces radioresistance. Rab12 up-regulates phosphorylation of Cdc25C(Ser216) after radiation, which is subsequently unable to dephosphorylate Tyr15 of Cdc2, thus maintains Cdc2 in an inactive form and induces more G2/M arrest. This prolonged G2/M arrest provides time for Rab12 to promote HRR. Our study reveals a molecular mechanism of radioresistance mediated by Rab12 in HPV-positive cervical cancer and helps to identify potential therapeutic targets to improve the efficiency of clinical treatment of cervical cancer.

## Data Availability Statement

The original contributions presented in the study are included in the article/**Supplementary Material**. Further inquiries can be directed to the corresponding author.

## Ethics Statement

The studies involving human participants were reviewed and approved by the Institute of Institutional Research Ethics of Shandong University. The patients/participants provided their written informed consent to participate in this study. Written informed consent was obtained from the individual(s) for the publication of any potentially identifiable images or data included in this article.

## Author Contributions

WFZ: design of study, manuscript preparation, and editing. YJH and YHT: data curation and manuscript preparation. WHZ and RJL: data curation. All authors contributed to the article and approved the submitted version.

## Funding

This research was funded by the National Natural Science Foundation of China, grant number 81571986 and the Natural Science Foundation of Shandong Province, grant number ZR2015HM084.

## Conflict of Interest

The authors declare that the research was conducted in the absence of any commercial or financial relationships that could be construed as a potential conflict of interest.

## Supplementary Material

Supplementary Figure 1 The abnormal expressions of Rab12 protein in human osteosarcoma, breast cancer, colon cancer, lung cancer, gastric cancer, and head and neck squamous cell carcinoma cells.
