# Supplementary figures and images for: Rab12 Promotes Radioresistance of HPV-Positive Cervical Cancer Cells by Increasing G2/M Arrest
Source: Front Oncol. 2021 Feb 25;11:586771. doi: 10.3389/fonc.2021.586771 (PMC7947205; doi:10.3389/fonc.2021.586771)

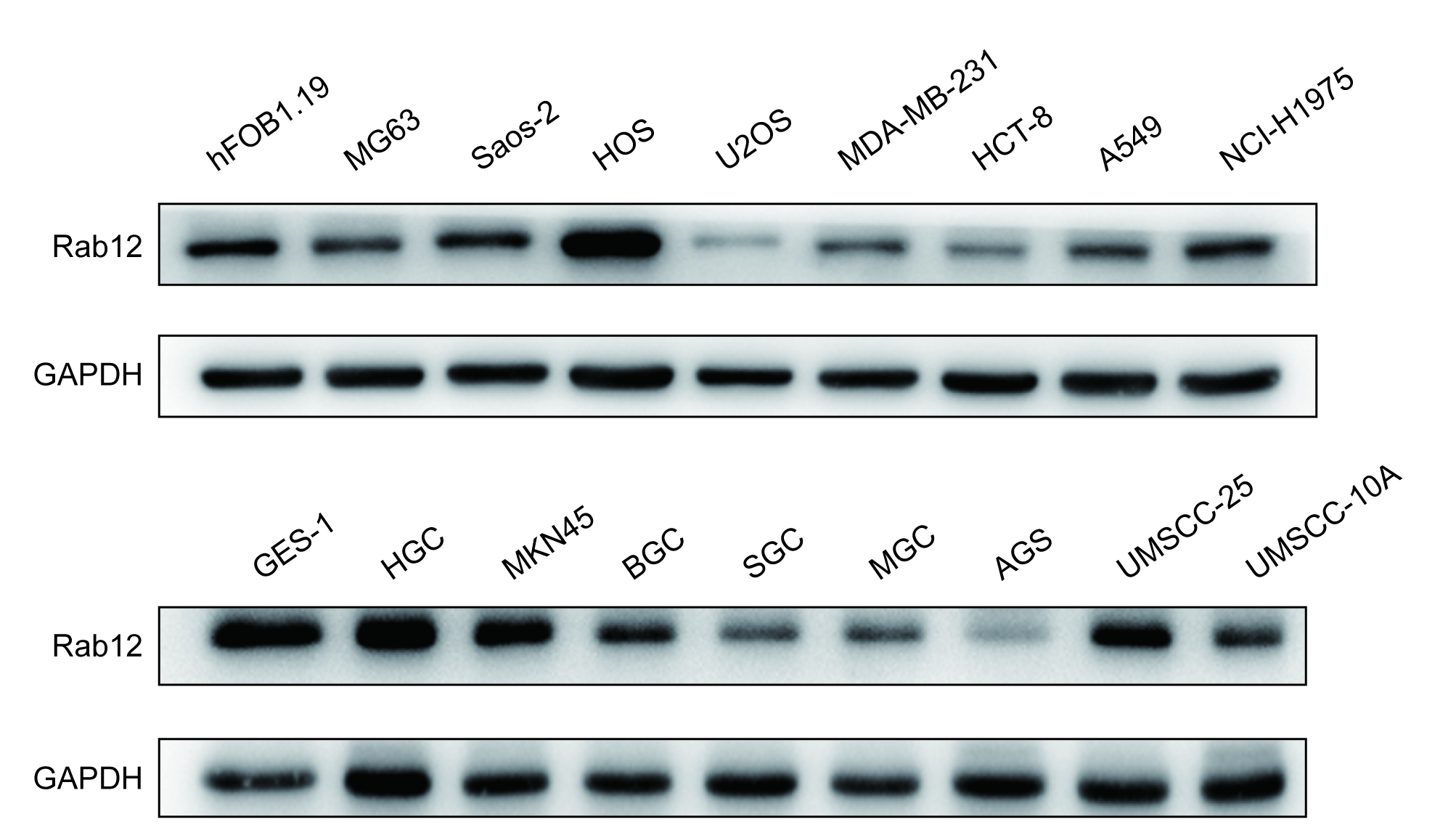

Supplement: Supplementary Figure 1 — The abnormal expressions of Rab12 protein in human osteosarcoma, breast cancer, colon cancer, lung cancer, gastric cancer, and head and neck squamous cell carcinoma cells. [file Image_1.jpeg]
